# Pain assessment in non-verbal children with neurocognitive impairment: a review on current tools, challenges, and clinical perspectives

**DOI:** 10.3389/fpain.2026.1751604

**Published:** 2026-03-31

**Authors:** Roaa Shaban

**Affiliations:** Department of Pediatrics, Faculty of Medicine, King Abdulaziz University, Jeddah, Saudi Arabia

**Keywords:** cognitive impairment, FLACC, management, NCCP, non-verbal children, pain assessment, pain assessment scales

## Abstract

Pain in non-verbal children with neurocognitive impairment (NCI) remains a complex and frequently under-recognized clinical challenge. Accurate pain assessment is essential for effective management, and is often hindered by non-verbal presentation, developmental adaptability, and the influence of sedation or illness. In response to these challenges, several observational tools have been developed to identify pain-related behaviors in non-verbal children. Among the most widely used tools is the Face, Legs, Activity, Cry, and Consolability (FLACC) scale, which was originally validated in young children and later adapted for use in children with communication barriers. Its revised version included additional behavioral descriptors tailored to children with cognitive impairments (CI). Similarly, the Non-Communicating Children's Pain (NCCP) Checklist was specifically developed for children with CI and provides a structured assessment across multiple behavioral domains. These tools have shown strong clinical utility and reliability, particularly in postoperative and critical care settings. Although both FLACC and the NCCP Checklists have proven effective, the latter is more designed to children with significant CI, suggesting a broader set of pain indicators. However, both tools require trained observers and clinical familiarity for optimal use. This review explores the strengths and limitations of these and other pain assessment tools, emphasizing the need for individualized approaches and validated instruments to ensure accurate pain recognition and treatment in pediatric populations, particularly those with NCI, including cases where critical illness further limits communication.

## Introduction

Non-verbal children with neurocognitive impairment are at high risk of inadequate pain assessment and management due to their limited ability to communicate effectively. Research studies focusing on pain in non-communicating and intellectually impaired children remain limited in strength, largely due to small sample sizes, heterogeneous patient populations, reliance on observational designs, and incomplete psychometric validation of assessment tools (1). According to the International Association for the Study of Pain (IASP), pain in children is defined as “an unpleasant sensory and emotional experience associated with actual or potential tissue damage or described in terms of such damage” (2). Because pain cannot be well-expressed in children lacking verbal communication, its evaluation remains a critical research focus pointed at improving understanding and enhancing their overall quality of life (QOL) (2). Pain considerably compromises QOL, often exerting a greater impact than physical disability alone. Repetitive or unrelieved pain can result in both short- and long-term neurodevelopmental consequences, including behavioral and cognitive alterations (3). When pain affects infants or children under two years of age—particularly those with neurological or cognitive impairments—it often persists, goes unrecognized, and remains poorly managed due to their inability to express discomfort. In contrast, children aged two years and above, who have developed basic verbal skills, are more suitable for self-report pain assessment scales (3, 4). Nonetheless, the efficiency of pain assessment tools largely depends on the child's cognitive ability to comprehend and communicate their pain accurately (4). Practical evidence indicates that self-report instruments such as the Faces Pain Scale–Revised (FPS-R) demonstrate strong validity and reliability in children aged 2–17 years, although convergent validity decreases below acceptable levels in those younger than seven years (5). For infants or children aged two years and above—with neurocognitive impairment—who cannot reliably self-report, behavioral assessment tools are preferred; however, no universally accepted “gold standard” exists due to limited psychometric validation (6).

Most national and international guidelines on infant pain management emphasize that systematic pain assessment is essential for achieving optimal pain control (7). Such assessment enables clinicians to identify when infants experience pain that warrants intervention and to prevent both under- and overtreatment with analgesics. The nature of pain in infants, compared with older children, makes assessment challenging (7). Care providers, especially nurses, must be trained to recognize and interpret pain signs in infants to determine when relief is needed. These signs fall into behavioral, physiological, hormonal, and neurophysiological domains, which are best evaluated together for greater accuracy (8). Consequently, a multidimensional approach to pain assessment is strongly recommended. For instance, full-term, healthy, and alert infants typically exhibit an upregulation of the fight-or-flight response, whereas preterm, critically ill, or deeply sleeping infants may display blunted responses (7). In a study of 109 nurses evaluating 158 non-verbal pain indicators, moaning, crying, and facial expressions during manipulation were rated as the most significant clues (9). Nurses also noted that the type of pain they relied on varied with the child's level of disability. They focused more on physiological signs in children with profound impairment, while social and emotional clues were emphasized in those with less severe disabilities, showing that a child's functional ability affects how pain is assessed (8).

Although several reviews have addressed pain assessment in specific pediatric populations, comprehensive syntheses focusing on non-verbal children across the spectrum of neurocognitive impairment remain limited. Existing reviews often concentrate on single age groups, clinical settings, or individual tools, restricting their clinical applicability. This review was conducted to integrate current evidence on observational pain assessment instruments, compare their strengths and limitations, and address practical challenges in implementation. By providing an updated, clinically oriented overview, this review also aims to support more accurate pain recognition and management in non-verbal children with neurocognitive impairment across diverse care settings.

## Methodology

To provide a structured and clinically oriented synthesis of the literature, we conducted a narrative review examining pain assessment tools used in non-verbal children with NCI. A comprehensive literature search was performed using PubMed, Scopus, and Web of Science, covering studies published up to October 2025. The search strategy combined relevant keywords and Boolean operators (AND/OR), including “pain assessment,” “non-verbal children,” “cognitive impairment,” “neurodevelopmental disorders,” “FLACC,” “Non-Communicating Children's Pain Checklist,” “behavioral pain scales,” “critical care,” and “pediatric pain.”

Eligible publications included original clinical studies, validation studies, observational research, and narrative or systematic reviews published in English that addressed pain assessment tools, psychometric properties, or clinical application in non-verbal pediatric populations. Studies focusing exclusively on self-report pain measures or adult-only populations were excluded. Article selection involved initial screening of titles and abstracts, followed by full-text review for relevance.

Selected studies were analyzed qualitatively, with emphasis on tool structure, target population, reliability, validity, feasibility, and clinical context of use. Findings were synthesized into thematic sections addressing infants, older children with neurocognitive impairment, and critically ill populations. As a narrative review, no formal quality scoring or meta-analytic techniques were applied.

## Results of narrative review

### Pain assessment in infants with or without neurocognitive impairment

The reviewed literature indicates that a considerable number of validated pain assessment scales for infants have been developed and implemented in both research and clinical practice (7). These instruments are generally categorized as unidimensional—focusing on behavioral or physiological parameters—or multidimensional, integrating several domains to enhance sensitivity and specificity (10). Across studies, an ideal infant pain assessment tool is consistently described as one capable of detecting painful situations with high sensitivity, differentiating pain from non-painful distress with adequate specificity, and demonstrating validity, reliability, and responsiveness to clinical interventions (11). However, evidence suggests variability in tool selection and methodological rigor. A systematic review of 352 infant pain studies reported that 16% of investigations used scales not validated for the specific population or pain type under study, highlighting a persistent gap between validation evidence and clinical application (12).

Among the most frequently utilized tools, the Premature Infant Pain Profile (PIPP/PIPP-R) was identified in 154 studies, followed by the Neonatal Infant Pain Scale (NIPS) in 84 studies, the Neonatal Facial Coding System (NFCS) in 33 studies, and the Douleur Aiguë du Nouveau-né (DAN) scale in 20 studies (12–15). Comparative analyses suggest that PIPP/PIPP-R demonstrates stronger psychometric robustness due to its incorporation of gestational age, behavioral state, facial expressions, and physiological indicators, producing a score from 0 to 21 (13). In contrast, NIPS emphasizes observable behavioral responses such as facial expression, crying, breathing patterns, limb movement, and arousal, with scores ranging from 0 to 7, which may limit multidimensional interpretation. NFCS focuses exclusively on facial muscle movements, scored by frequency and intensity (15, 16), potentially narrowing its scope in complex clinical situations. DAN integrates facial expression, limb motion, and vocal response (12), yet comparative literature suggests variation in responsiveness depending on procedural context.

Technological advancements have attempted to address subjectivity inherent in observational tools. The ABC analyzer, a computer-based crying frequency assessment system, showed significant correlation with DAN scores during heel-prick procedures (17), indicating potential adjunctive validity. Furthermore, a multimodal sensor-based machine-learning system incorporating facial electromyography, ECG, electrodermal activity, oxygen saturation, and EEG demonstrated promise for observer-independent monitoring (18). Nevertheless, these technologies remain investigational, and large-scale validation studies are limited.

Glenzel et al., in a systematic review of 25 tools assessing pain, stress, and behavior in neonatal intensive care settings, reported that PIPP and PIPP-R exhibited the highest levels of validity, reliability, and measurement quality (19). Overall, the literature supports the availability of validated infant tools; however, inconsistencies in validation populations, contextual application, and methodological rigor remain evident.

### Pain assessment in older children with neurocognitive impairment

Older children with neurocognitive dysfunction also experience difficulty expressing or describing their pain. Some neurological diseases in childhood are frequently accompanied by cognitive impairment (CI), encompassing a wide range of conditions such as intellectual disability and global developmental delay, in which intellectual functioning and adaptive skills are significantly below the expected level for age (20). CI may result from diverse etiologies, including cerebral palsy (CP), genetic syndromes, traumatic brain injury, or neurodegenerative disorders, yet there remains no consensus on the most appropriate pain assessment tool in these contexts.

Among these conditions, CP has been the most extensively studied with respect to both pain prevalence and assessment. Reported prevalence rates vary considerably, ranging from 15% to 75%, with higher rates observed in non-ambulatory adults, reaching approximately 80% (21, 22). This wide range suggests substantial heterogeneity in study design, severity classification, and assessment methodology. Mechanistically, pain in CP is attributed to multiple contributors, including muscle overuse, involuntary movements, prolonged immobilization, and abnormal joint compression secondary to muscle imbalance across joints (2). The literature indicates that pain is highly prevalent in partially or completely non-verbal children and often occurs daily, particularly during physiotherapy sessions (1). Notably, its occurrence appears more strongly associated with child-specific clinical factors—such as age and spastic quadriplegia—than with psychosocial influences, suggesting that structural and motor severity play a dominant role in pain burden (23). Children with profound motor damage demonstrate greater musculoskeletal pain frequency and intensity, further reinforcing the link between functional limitation and pain severity (23).

Assessing pain in non-verbal children with CP remains a major challenge. Non-verbal children with CP or children with severe CI have consistently been identified as being at high risk for experiencing pain compared to their typically developing peers, particularly during routine caregiving tasks (24, 25). In addition, they are frequently vulnerable to chronic medical conditions that not only aggravate pain but also often necessitate surgical interventions (20).

From a clinical standpoint, the literature emphasizes the importance of distinguishing between children with CI who retain the ability to articulate pain and those who do not. Studies demonstrate that a substantial proportion of children with CP who are able to self-report experience recurrent pain, highlighting the chronic and persistent nature of discomfort in this population (26). In the hemiplegic variant of CP, pain is commonly associated with reduced quality of life (QOL), affecting participation in activities, sleep quality, energy levels, and mood (27). A Swedish study by Westbom et al. involving 497 children with CP reported that 37% experienced pain, with the highest prevalence observed in dyskinetic forms and in children with severe functional limitations. Pain most frequently involved the feet and knees (28). These findings underscore that pain distribution patterns may be anatomically predictable in certain subtypes, yet assessment tools must still account for communication variability.

In children diagnosed with autism spectrum disorders (ASD), pain expression is notably heterogeneous. Most of children may exhibit both hypersensitivity and hyposensitivity to pain, and in some cases, may show no noticeable behavioral signs of discomfort (29). Children with ASD typically demonstrate qualitative deficits in language, expressing pain through repetitive or stereotyped behaviors that can be easily misinterpreted by others (29). Initial studies in this population noted a lack of obvious pain responses—such as facial grimacing or withdrawal reflexes—despite physiological evidence of distress, including elevated heart rates and increased blood endorphin concentrations during painful stimuli when compared to neurotypical controls (20). This dissociation between physiological response and observable behavior complicates reliance solely on behavioral cues and highlights the importance of structured assessment tools.

A number of other common disabilities with onset in childhood are frequently associated with significant experiences of pain. For instance, in individuals with spina bifida, the prevalence of pain during young adulthood has been reported to range between 30% and 40% (30). Similarly, among survivors of childhood central nervous system (CNS) cancers, the prevalence of pain in young adulthood is estimated at 35% with a markedly increased relative risk—approximately 7.9 times higher—when compared to their unaffected siblings (31).

Importantly, approximately 50% of children with borderline, mild, or moderate CI are capable of using numerical pain-rating scales to communicate discomfort (32). Thus, when feasible, self-report remains the preferred and most effective method for pain assessment (33). However, despite improvements in pain management practice, children with CI continue to experience significant gaps in recognition, particularly in preoperative contexts compared with postoperative settings (34, 35). This discrepancy may reflect differences in monitoring intensity and structured assessment during hospitalization. Comprehensive pain assessment should incorporate careful monitoring of physiological signs and behavioral manifestations potentially linked to pain. In this process, the role of family caregivers and healthcare providers becomes critical. Some mothers become highly practiced in identifying their child's pain without professional intervention (36). Conversely, caregivers have been observed to sometimes overestimate symptoms in acute pain scenarios while underestimating them in chronic pain conditions (37).

Adults who have grown up with childhood-onset disabilities, particularly those involving neurocognitive impairments, also encounter persistent or recurrent pain as they grow. Due to communication barriers often present in this population, it becomes essential for caregivers and healthcare providers to rely on the observation and interpretation of non-verbal keys as potential signs of pain (38). However, this reliance on indirect indicators presents significant challenges, often resulting in misinterpretations that contribute to delays or inaccuracies in both diagnosis and treatment. Although pain evaluation in children with neurological impairments has received considerable attention in the literature, there remains a lack of comprehensive evidence-based guidelines tailored to pain assessment and management in adults with similar conditions.

### Pain assessment tools in non-verbal children with neurocognitive impairment

Since the early 2000s, numerous tools, checklists, and scales have been introduced to assess pain in children, each including different sets of observable pain signs (24, 39, 40). Despite these advancements, no single tool has demonstrated superior accuracy over the others, and none have been formally validated for use in adult populations. Additionally, there is currently no universally accepted or standardized tool specifically recommended for assessing pain in children with non-verbal communication and adults who have childhood-onset disabilities who face significant communication challenges. Studies addressing pain assessment in adults with such disabilities remains sparse and underdeveloped (38).

As previously discussed, assessing pain in children with neurocognitive impairments presents considerable challenges, primarily due to the presence of multiple, overlapping sources of pain and the frequent occurrence of communication barriers that complicate pain expression. In an effort to address this gap, Pizzinato et al. conducted a meta-analysis focused on evaluating the reliability of pain assessment tools in children with neurocognitive impairment, specifically for postoperative pain contexts (20). Their review encompassed a wide range of databases and included studies published from 2000 to 2022. Out of the literature screened, 12 studies met the inclusion criteria, representing a sample of 586 children with a mean age of 10 years. Among the tools assessed, the Non-Communicating Children's Pain Checklist–Postoperative Version (NCCPC-PV) demonstrated an intraclass correlation coefficient (ICC) of 0.76, while the Face, Legs, Activity, Cry, Consolability (FLACC) scale showed a slightly higher ICC of 0.87, suggesting good reliability in clinical use (20). Despite notable heterogeneity across the studies, both the NCCPC-PV and the FLACC emerged as the most valid and consistently reliable tools for evaluating postoperative pain in children with neurocognitive impairment (20).

#### Non-communicating children's pain checklist–postoperative version (NCCPC-PV)

The *Non-Communicating Children's Pain Checklist* (NCCPC) is a 27-item observational tool specifically developed for assessing pain in children with CI who are unable to communicate verbally (24). The scale demonstrates a reasonable ability to distinguish between painful and non-painful episodes. To improve specificity, the postoperative version (NCCPC-PV) was introduced, which eliminates items related to eating and sleeping—two domains that could increase the risk of false positives. However, both versions can be used for non-verbally communicated children with CI. NCCPC-PV organizes observed behaviors into six distinct categories: vocal expressions (such as moaning or crying), social interactions (withdrawal or seeking comfort), facial expressions (e.g., grimacing, frowning), body and limb movements (such as rigidity or protective gestures), physiological signs (like altered breathing or skin color), and activity level (restlessness or decreased movement) (24, 41). Each behavior is assessed based on its frequency or intensity during a defined observation period, commonly in the postoperative setting. A score equal to or greater than 11 is considered indicative of clinically significant pain, showing a high sensitivity level—detecting pain in up to 90% of affected children. Reported psychometric properties of the NCCPC-PV include intraclass correlation coefficients ranging from 0.63 to 0.88 across studies, with sensitivity reported up to 90% for detecting postoperative pain at a cutoff score ≥11. Internal consistency has been reported as good, with Cronbach's alpha values generally exceeding 0.70 (24, 25, 41).

The validity of the scale has been examined in multiple cultural and linguistic studies. For instance, Zanchi et al. evaluated the Italian adaptation of the NCCPC-PV (I-NCCPC-PV) in a cohort of 40 non-verbal children with intellectual disabilities (40). The study found significant behavioral differences between pain and calm states, as well as fair to good inter-rater reliability and concordance, thus supporting its clinical validity. Similarly, a Brazilian study conducted in 2020 involved the translation and cultural adaptation of this assessment tool into Portuguese through a process involving expert review and field testing with healthcare professionals and caregivers (42). The results showed semantic clarity and cultural suitability for pain assessment in non-verbal children with intellectual disabilities. While other studies—such as those conducted in Sweden—have also confirmed the tool's reliability in various settings, its application remains largely limited to children with severe CI (34, 41). This narrow focus may hinder the generalizability of its findings to populations with mild to moderate CI, where pain expression might differ in nature or intensity. Furthermore, Voepel-Lewis et al. observed that the interpretation of NCCPC-PV scores often lacks consistency when compared to other pain measures (43). As a result, clinicians ranked the tool as significantly less feasible for routine use in clinical settings, particularly when compared with more flexible assessment scales (43, 44). A similar conclusion was drawn by Pizzinato et al., whose meta-analysis emphasized the practical challenges of applying the NCCPC-PV in routine clinical care (20). While the tool demonstrated acceptable levels of reliability in research settings, its use in daily healthcare practice was limited by several factors—such as the time-intensive nature of the checklist, the need for observer training, and the potential for subjective variation in interpreting behavioral signs. These constraints reduce the tool's feasibility and delay its adoption in clinical environments, particularly when compared to more streamlined and flexible instruments. Salabura et al. also reviewed 14 studies on pain assessment in children with ASD, underlining the need for personalized approaches (45). While behaviors varied, the NCCPC proved useful. Parent involvement and tailored assessment methods were strongly recommended.

#### Face, legs, activity, cry, consolability (FLACC)

The Face, Legs, Activity, Cry, Consolability (FLACC pain scale) is a well-established behavioral assessment tool validated for use in children, particularly for children up to 16 years of age who are unable or unwilling to verbally communicate due to neurocognitive impairment (46). Notably, it is designed to assess both pain intensity and psychological distress (47). FLACC was initially developed and validated to assess acute postoperative pain in healthy children aged 2 to 7 years; however, its use has been extended to a broader range of clinical scenarios, including the assessment of preoperative pain (48, 49). The scale is composed of five distinct behavioral categories—facial expression, leg movement, activity, cry, and consolability—each rated on a 3-point scale from 0 to 2. The cumulative score ranges from 0 (indicating a relaxed and comfortable state) to 10 (reflecting severe pain or distress). For example, behaviors such as grimacing, frequent restlessness, or persistent crying correspond to higher scores in their respective domains. This structured approach offers clinicians a fast, objective, and consistent method for evaluating pain in children who cannot self-report. To enhance its applicability in children with CI, a revised version of the FLACC—r-FLACC—was developed. This version includes expanded behavioral descriptors such as verbal outbursts, tremors, spasticity, jerking movements, and irregular breathing patterns, thereby capturing a broader range of pain-related behaviors often observed in children with CI (34). These additions significantly improved reliability and sensitivity across all scoring categories. Psychometric evaluation of the FLACC and revised FLACC scales demonstrated strong inter-rater reliability, with intraclass correlation coefficients ranging from 0.82 to 0.97, and high construct validity when compared with parental and clinician pain ratings (34). The revised FLACC showed improved sensitivity in children with cognitive impairment by incorporating individualized behavioral descriptors. Interestingly, during the validation process, some parents reported that the absence of behavioral responses—such as reduced expression or interaction—was a key sign of pain in their children, suggesting that these more nuanced clues were critical for improving the accuracy of r-FLACC (34). The Korean version of the FLACC (K-FLACC) scale was also re-developed to assess pain in patients with cognitive dysfunction (50). Validated in 88 individuals, it showed strong correlations with numeric rating scale (NRS) and face pain scale (FPS) scores, and excellent test reliability.

Further insight into the comparative use of pain assessment tools is provided by Pizzinato et al. (20). Their findings highlighted occasional discrepancies in pain scoring between researchers and caregivers, particularly when distinguishing between painful and non-painful conditions. However, these differences were not statistically significant, suggesting that both groups were largely consistent in their assessments across the two tools. A detailed comparison of the NCCPC-PV and FLACC scales, including structure, scoring, and observed outcomes, is presented in Table 1, providing a comprehensive overview of their strengths and limitations. Overall, while both tools demonstrate good reliability and validity, the FLACC scale offers greater feasibility and ease of use in routine clinical settings, whereas the NCCPC-PV provides higher specificity for children with severe cognitive impairment but requires more time and observer training.

**Table 1:** Comparison between FLACC and NCCPC-PV pain assessment scales.

Characteristic	FLACC	NCCPC
Definition	Behavioral pain assessment tool for children unable to communicate effectively, including infants and young children.	Observational checklist developed specifically for assessing postoperative or preoperative pain in children with CI.
Tool Type	Quick behavioral observation scale.	Comprehensive caregiver-based observational questionnaire.
Contents/Items	5 behavioral categories: Face, Legs, Activity, Cry, Consolability.	27 behavioral indicators across 6 domains: Vocalizations, Social interactions, Facial expressions, Body and limb movements, Physiological signs, and Activity level.
Scoring System	Each category scored 0–2, total 0–10. Higher scores = greater pain intensity.	Each item scored 0–3 (not at all to very often); total score 0–81. ≥11 indicates clinically significant pain.
Assessment Method	Observation by nurses or clinicians during care or procedures.	Completed by caregivers familiar with the child, based on observed behaviors postoperatively.
Target Population	Infants and young children (2 months–7 years), including postoperative and ventilated patients.	Non-verbal or cognitively impaired children (e.g., cerebral palsy, developmental delay) unable to self-report pain.
Time to Administer	Rapid – typically 1–2 min.	Longer – approximately 10–15 min.
Primary Setting	Pediatric wards, ICU, and postoperative recovery.	Postoperative and home care for children with developmental or intellectual disabilities.
Impact/Clinical Use	Simple, reliable, and suitable for repeated bedside use; detects acute procedural pain.	Sensitive to subtle behavioral cues in cognitively impaired children; improves caregiver recognition of pain.
Reliability & Validity	High interrater reliability validated for acute Pre- and Post-operative pain.	High validity and reliability for chronic pre-and postoperative pain.
Advantages	Quick, easy, requires minimal training, suitable for clinical routines.	Tailored for non-verbal children, captures complex pain behaviors, sensitive to subtle changes.
Limitations	Less sensitive to chronic or atypical pain behaviors in cognitively impaired children.	More time-consuming; requires caregiver familiarity and clinical context.
Accessibility	Freely available and widely used in hospitals worldwide.	Freely available in published literature; less commonly used outside specialized pediatric centers.
Overall Summary	Ideal for general pediatric and ICU pain assessment where rapid observation is needed.	Best suited for children with cognitive or communication impairments requiring detailed behavioral evaluation.

CI, cognitive impairment; FLACC, face, legs, activity, cry, consolability; NCCP, non-communicating children's pain checklist; ICU, intensive care unit.

#### Statistical comparison of NCCPC-PV and FLACC in children with NCI

Comparative evidence suggests that both NCCPC-PV and FLACC demonstrate acceptable psychometric performance; however, differences emerge in reliability ranges, sensitivity thresholds, and feasibility. Reported intraclass correlation coefficients (ICC) for NCCPC-PV range from 0.63 to 0.88 across studies, indicating moderate to good inter-rater reliability, with sensitivity reported up to 90% at a cutoff score ≥11 for postoperative pain detection (24, 25, 41). Internal consistency is generally adequate, with Cronbach's alpha values exceeding 0.70. In contrast, FLACC and its revised version (r-FLACC) demonstrate consistently higher ICC values ranging from 0.82 to 0.97, suggesting stronger inter-rater agreement and measurement stability (34). The meta-analysis by Pizzinato et al. (20) further supports this distinction, reporting ICC values of 0.76 for NCCPC-PV and 0.87 for FLACC in postoperative settings, while acknowledging heterogeneity across studies. Although both tools show good validity, FLACC demonstrates greater feasibility and efficiency in clinical practice due to its shorter structure and ease of scoring. Conversely, NCCPC-PV may offer enhanced behavioral specificity in children with severe cognitive impairment but requires more time and observer training, potentially limiting routine implementation.

#### Other assessment tools in neurocognitive impairment

In addition to widely used tools like NCCPC and FLACC, other pain assessment tools have been studied in the literature for evaluating pain in patients with CI. One such tool is the *Nursing Assessment of Pain Intensity* (NAPI), which is considered one of the most user-friendly and accessible tools for assessing mild, moderate, and severe pain in cognitively impaired patients. Although it was not originally developed for children with CI, it has been found to be easily applicable by caregivers and clinicians due to its simple structure and minimal reliance on prior familiarity with the child's typical behavior (51, 52). The NAPI has demonstrated excellent reliability (46). This scale relies on the observation of both behavioral and physiological signs, including facial expressions, body movements, muscle tension, vocalizations such as crying or moaning, and autonomic signs like changes in heart rate or respiratory pattern. Each domain is scored from 0 (no sign of pain) to 2 (strong indicators of pain), producing a cumulative score from 0 to 10. Higher scores are interpreted as reflecting more severe pain levels.

*Children's Hospital of Eastern Ontario Pain Scale* (CHEOPS) is another pain assessment tool that evaluate pain through six behavioral domains: cry, facial expression, verbal response, torso movement, touch response, and leg position. Each is rated on a 0–2 scale, with total scores ranging from 0 to 10, where higher values indicate more intense pain. In a study involving 69 Thai children following surgery, both nurses and parents were able to effectively use the CHEOPS scale with high agreement and correlation in pain rating (53). Notably, the study found that parental education level had no significant effect on the accuracy of scoring, and the tool itself was straightforward to apply after brief training. In contrast, the *Distress and Emotional Scale Score* (DESS) was reported to be significantly more complex in practice. It requires a higher degree of observer familiarity with the child's usual behavior, making it less feasible for use by individuals with limited training or unfamiliarity with the child—an issue particularly relevant in clinical settings (51).

A significant contribution by Terstegen et al. involved semi-structured interviews with parents and healthcare professionals to identify over 200 potential pain signs for children with CI (54). After excluding items unsuitable for clinical use, 138 behaviors remained and were evaluated postoperatively against the *Visual Analogue Scale* (VAS). Statistical analysis revealed 23 indicators with the highest correlation to pain, forming the basis of a focused, clinically practical questionnaire. This refined tool improved postoperative pain assessment accuracy in children with CI (54).

### Pain assessment in critically ill children: contextual considerations for non-verbal assessment

Although the primary focus of this review is children with neurocognitive impairment (NCI), it is important to acknowledge that a subset of critically ill children without baseline NCI may also become functionally non-verbal due to sedation, mechanical ventilation, reduced consciousness, or severe medical instability. In these circumstances, the challenges of pain assessment closely resemble those encountered in children with NCI, particularly the reliance on behavioral observation and the difficulty of distinguishing pain-related behaviors from distress, illness severity, or treatment effects. The inclusion of evidence from pediatric intensive care settings is therefore intended to illustrate overlapping methodological challenges in non-verbal pain assessment rather than to redefine the population of interest. This distinction emphasizes that critical illness is not equivalent to neurocognitive impairment but represents a clinical context in which similar assessment limitations arise.

In the intensive care unit (ICU), critically ill children are frequently exposed to painful procedures and conditions that require accurate assessment and timely management. A major challenge in this setting is that many of these children are unable to communicate verbally—either due to sedation, unconsciousness, mechanical ventilation, or pre-existing neurocognitive impairment (55, 56). Pain assessment in this population requires specialized expertise, as clinicians must differentiate between pain-related behaviors and manifestations resulting from cognitive, emotional, or situational factors, including mechanical ventilation and pharmacologic sedation. Stevens et al. emphasized the difficulty of distinguishing pain from symptoms related to illness progression, treatment side effects, sedation, or immobility, all of which may mimic or obscure pain expression (57).

In pediatric ICUs (PICUs), physiological parameters such as heart rate, blood pressure, and oxygen saturation are often used as indirect indicators of pain. However, their reliability is limited due to the frequent administration of inotropes, chronotropes, sedatives, and other vasoactive medications that may significantly alter these variables independent of pain (57). This limitation reinforces the importance of validated behavioral tools in non-verbal patients.

In response to these assessment challenges, validated instruments have been developed for sedated or ventilated children. The COMFORT scale evaluates pain and distress using eight indicators, including alertness, calmness, respiratory response, physical movement, muscle tone, facial tension, heart rate, and blood pressure, with each item rated from 1 to 5 and total scores ranging from 8 to 40; scores ≥27 indicate increased pain or distress (58–60). The COMFORT-B scale, a revised behavioral-only version excluding physiological parameters, focuses on six behavioral domains and yields total scores ranging from 6 to 30. By eliminating physiologic variables that may be pharmacologically influenced, COMFORT-B demonstrates improved applicability in sedated or ventilated patients and has shown strong reliability in critically ill children who cannot self-report (58–60).

Gascony et al. evaluated the Behavioral Pain Scale (BPS) in 84 sedated, intubated, mechanically ventilated pediatric ICU patients. Compared with COMFORT-B and the Numeric Rating Scale (NRS), BPS demonstrated strong internal consistency (*α* = 0.86), high concurrent validity, and good discriminant validity based on receiver operating characteristic (ROC) analysis, supporting its reliability and feasibility in PICU settings (60). These findings indicate that structured behavioral scales can maintain psychometric robustness even in complex critical care environments. Mattsson et al. conducted a qualitative phenomenographic study involving 17 PICU nurses assessing non-verbal children aged 2–6 years (59, 60). Three principal categories of pain expression emerged: changes in physiological signs, muscular tension, and behavioral alterations. These findings underscore that pain recognition in critically ill children—similar to children with NCI—requires individualized, holistic, and patient-centered observation rather than reliance on isolated indicators.

Additionally, Johanson et al. compared the COMFORT-B scale with the modified FLACC scale in ventilated PICU patients (59). Both instruments demonstrated strong correlation with nurses' clinical pain ratings and high inter-rater reliability. However, COMFORT-B showed greater sensitivity in identifying sedation levels, whereas the FLACC scale more accurately reflected changes in pain following analgesic administration. These comparative findings suggest that tool selection may depend on the primary clinical objective—sedation monitoring vs. analgesic response evaluation.

Overall, while critically ill children without baseline NCI represent a distinct population, the assessment challenges encountered in PICU settings provide relevant methodological insight into structured behavioral pain evaluation in non-verbal pediatric patients. The evidence presented here therefore supports broader understanding of behavioral pain assessment principles applicable to children with neurocognitive impairment who similarly rely on observer-based tools.

## Limitation

This review has several limitations. First, it is narrative in nature and does not follow a formal systematic review or meta-analytic methodology. Second, the included studies show considerable heterogeneity in study design, sample size, clinical setting, and reported psychometric outcomes, limiting direct quantitative comparison between assessment tools. In addition, many tools were validated in specific populations or contexts, which may restrict generalizability. Despite these limitations, this review provides a focused, clinically relevant synthesis of commonly used pain assessment tools for non-verbal children with neurocognitive impairment.

## Conclusions

This narrative review examined the current evidence on pain assessment tools for non-verbal children with NCI, highlighting both methodological strengths and persistent limitations across available instruments. The literature consistently demonstrates that children with NCI experience frequent and often under-recognized pain, particularly in postoperative and routine care contexts. Among validated behavioral tools, the FLACC and revised FLACC scales show strong inter-rater reliability and feasibility in clinical practice, with intraclass correlation coefficients ranging from 0.82 to 0.97, supporting their use in diverse pediatric settings. In contrast, the NCCPC-PV demonstrates high sensitivity (up to 90% at a cutoff ≥11) and good internal consistency, making it particularly useful for children with severe cognitive impairment, although its time-intensive structure may limit routine feasibility. Evidence from comparative studies suggests that no single tool demonstrates universal superiority; rather, tool selection should be guided by cognitive severity, clinical setting, and assessment objectives. Despite advances, heterogeneity in validation populations, reliance on observer interpretation, and limited data in adult survivors of childhood-onset disabilities remain important gaps. Future efforts should focus on standardizing implementation strategies, improving caregiver training, and integrating individualized behavioral descriptors and technology-assisted monitoring to enhance accuracy and clinical applicability.
